# Supplementary material for: The Pseudomonas aeruginosa RpoH (σ32) Regulon and Its Role in Essential Cellular Functions, Starvation Survival, and Antibiotic Tolerance
Source: Int J Mol Sci. 2023 Jan 12;24(2):1513. doi: 10.3390/ijms24021513 (PMC9866376; doi:10.3390/ijms24021513)
Supplement: Supplementary file 1 [file ijms-24-01513-s001.zip › ijms-2055647-supplementary.pdf]

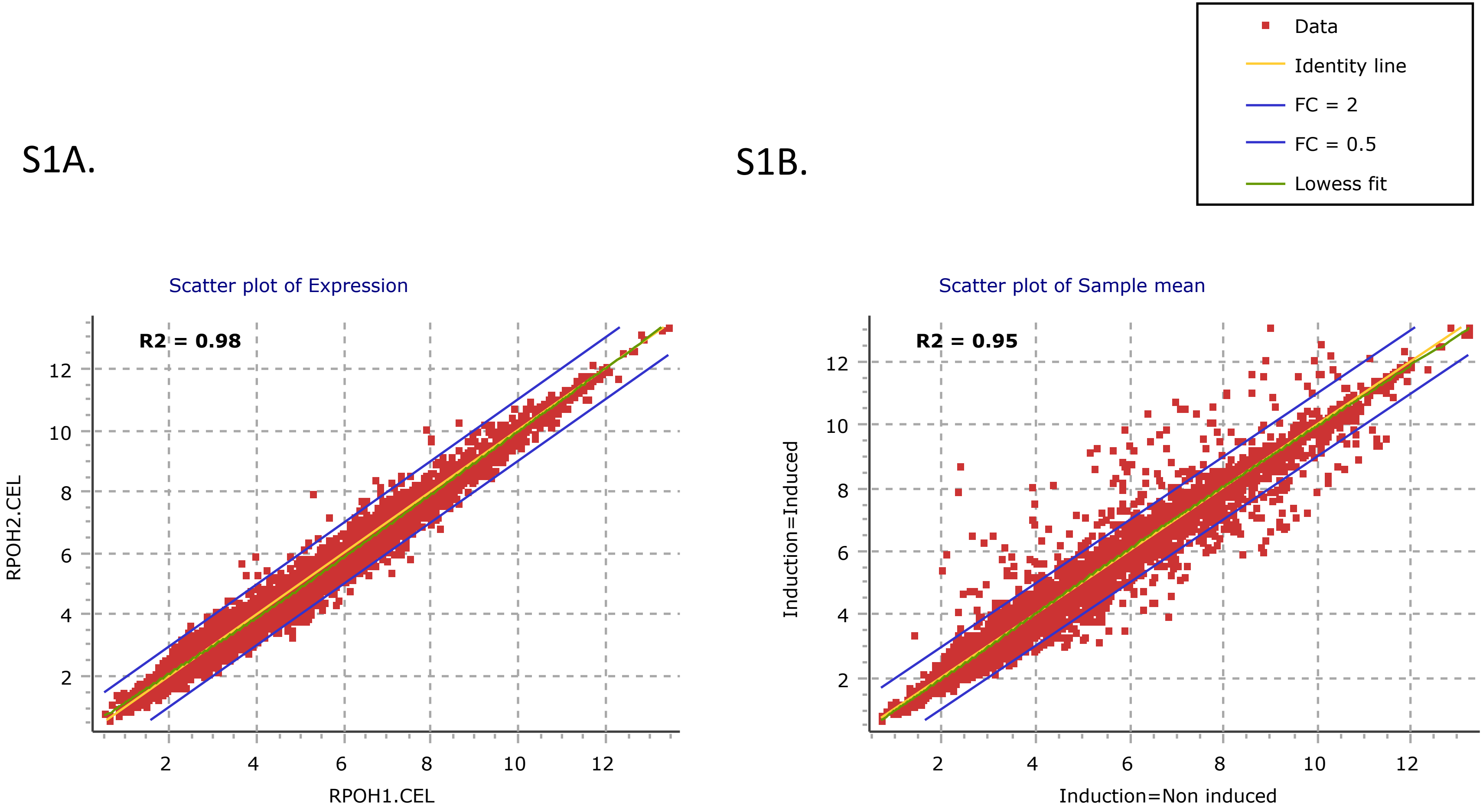

Figure S1. Scatter plots of *P. aeruginosa* Δ*rpoH* (P_{BAD}-*rpoH*) transcriptome expression data. (A) Independent biological replicates showed highly reproducible gene expression values; $R^2 = 0.98$ in the example above. (B) Arabinose induction of *rpoH* resulted in 258 genes with significantly different expression (at $p < 0.05$ and > two-fold change) compared to the non-induced controls. Three independent biological replicates were performed.

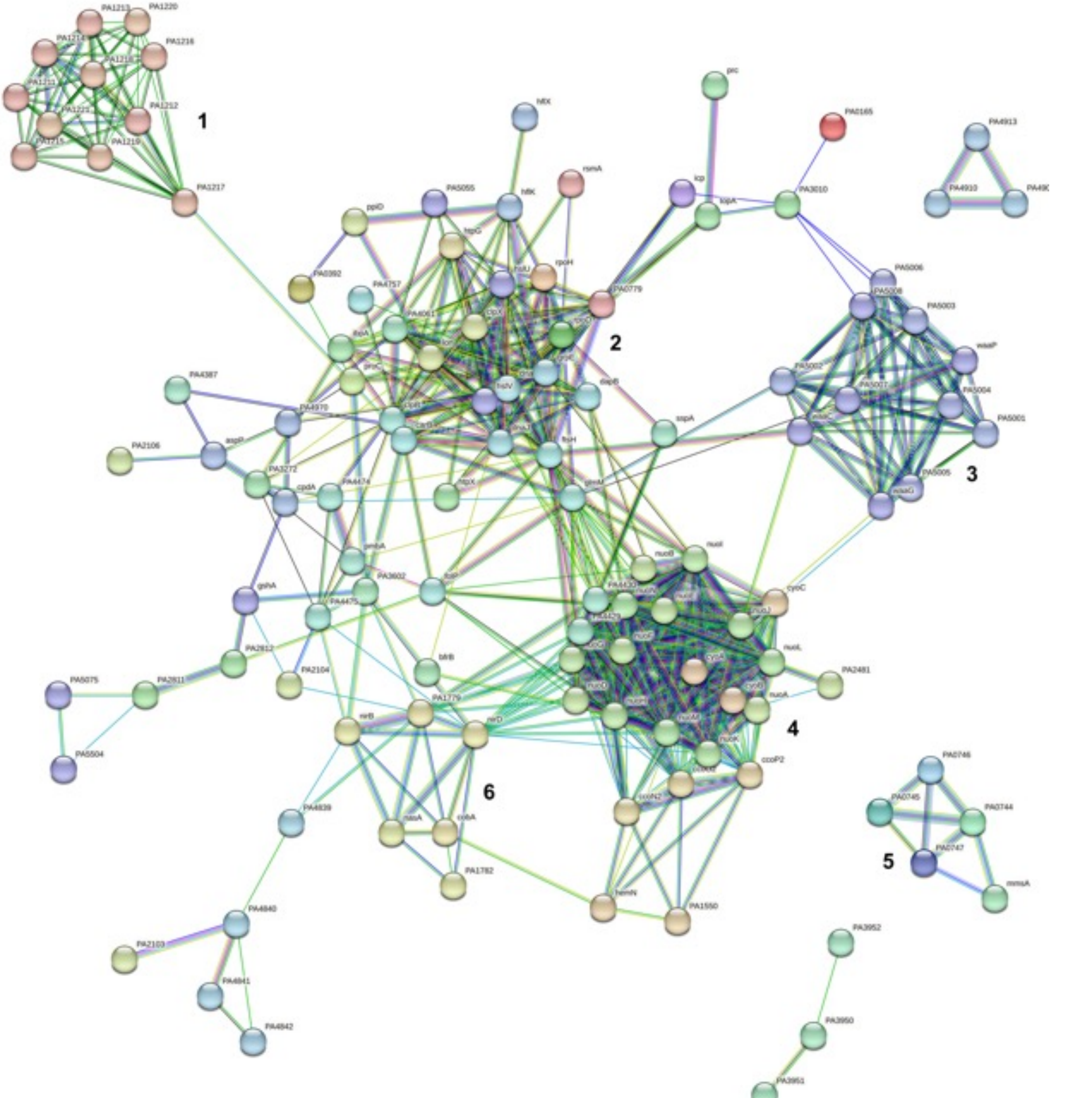

Supplementary Figure S2. *STRING analysis of genes upregulated by RpoH.* Genes with no functional connections, and those connected only to one partner, are not shown for clarity. General functional assignments of labeled clusters are as follows: 1) Non-ribosomal peptide antibiotic biosynthesis; 2) Chaperones and proteases; 3) Cell wall and membrane synthesis and modification; 4) NADH dehydrogenase complex *nuo*; 5) Isomerases / dehydrogenases involved in redox reactions; 6) Assimilatory nitrate reduction.

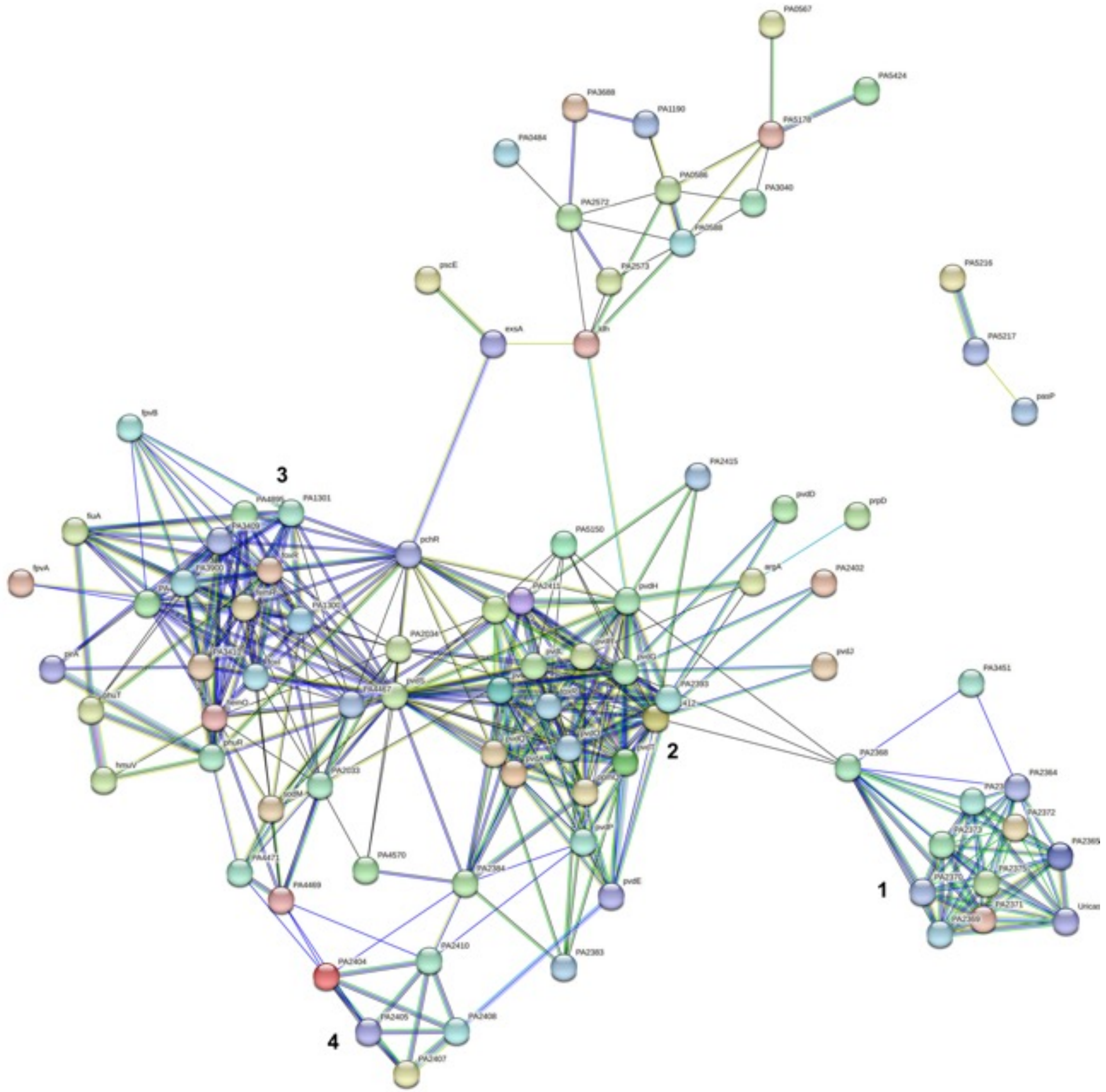

Supplementary Figure S3. *STRING analysis of genes downregulated by RpoH.* Genes with no functional connections, and those connected only to one partner, are not shown for clarity. General functional assignments of labeled clusters are as follows: 1) Members of type VI secretion system and uncharacterized; 2) Pyoverdine synthesis and export; 3) Multiple sigma factors; 4) Iron binding and reduction, *fpvGHJKCDEF* operon.

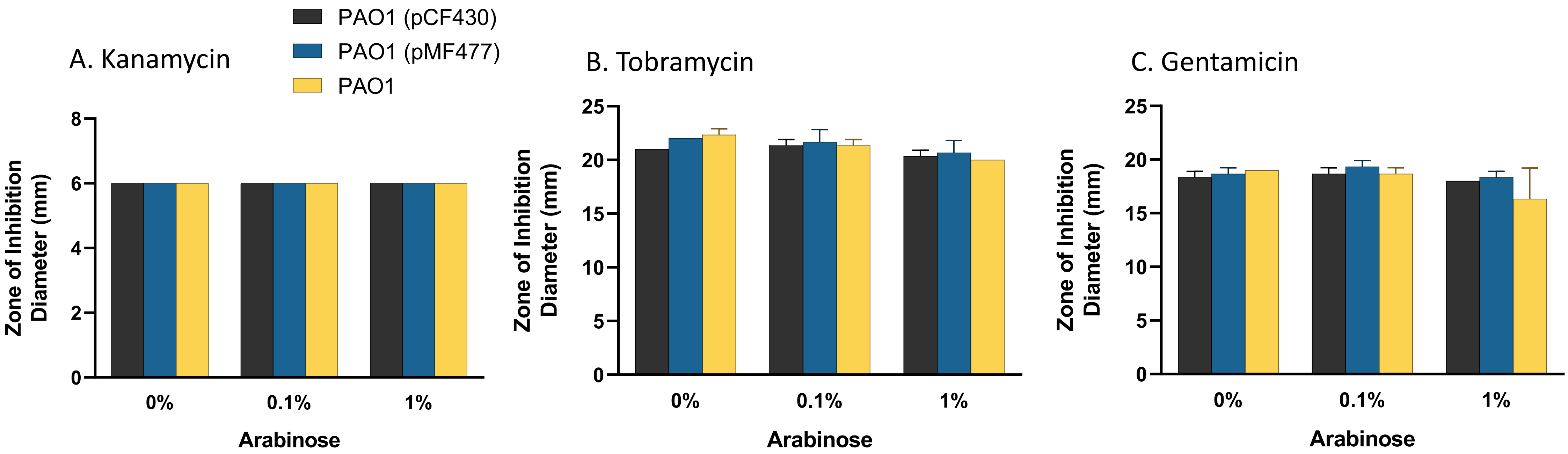

Figure S4. Antibiotic sensitivities of *P. aeruginosa* PAO1, *P. aeruginosa* PAO1 (pCF430), and *P. aeruginosa* PAO1 (pMF477) assayed by the disk diffusion assay, to determine the effect of plasmids used in this study on antibiotic tolerance of *P. aeruginosa*. The results show the mean and standard deviation of three independent biological replicates. The results show no effects on the zone of clearing around the aminoglycoside disks due to the plasmids used in this study.

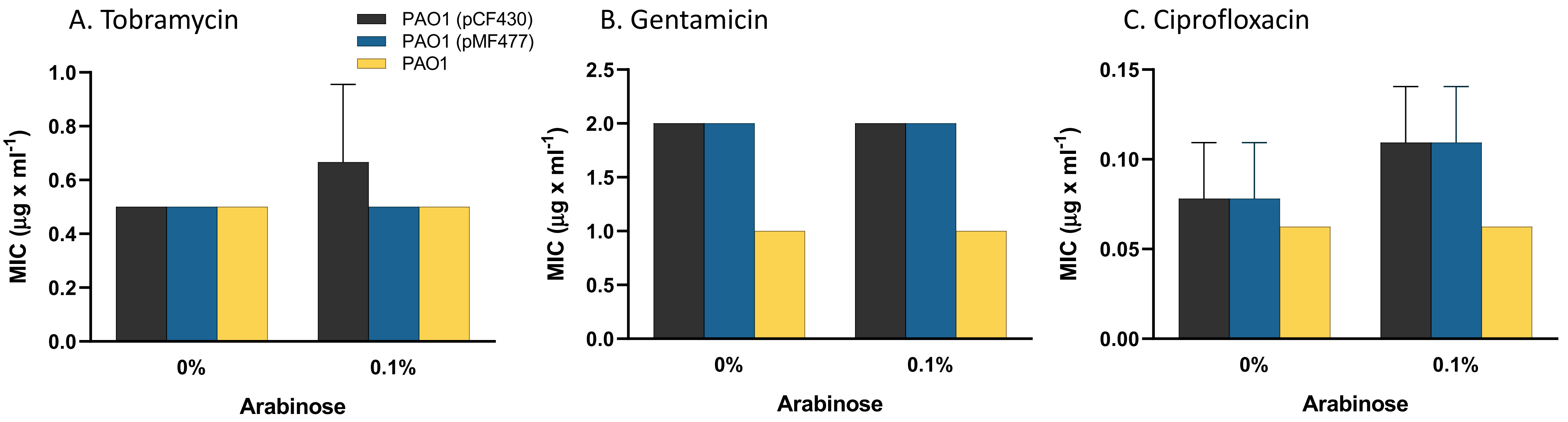

Figure S5. Antibiotic sensitivities of *P. aeruginosa* PAO1, *P. aeruginosa* PAO1 (pCF430), and *P. aeruginosa* PAO1 (pMF477) assayed as minimum inhibitory concentration (MIC), to determine the effect of plasmids used in this study on antibiotic tolerance of *P. aeruginosa*. The results show the mean and standard deviation of three independent biological replicates. The results show that the vector control and the plasmid containing *rpoH* (pMF477) resulted in an increased MIC to gentamicin, but no significant effect on the MIC to tobramycin or ciprofloxacin.

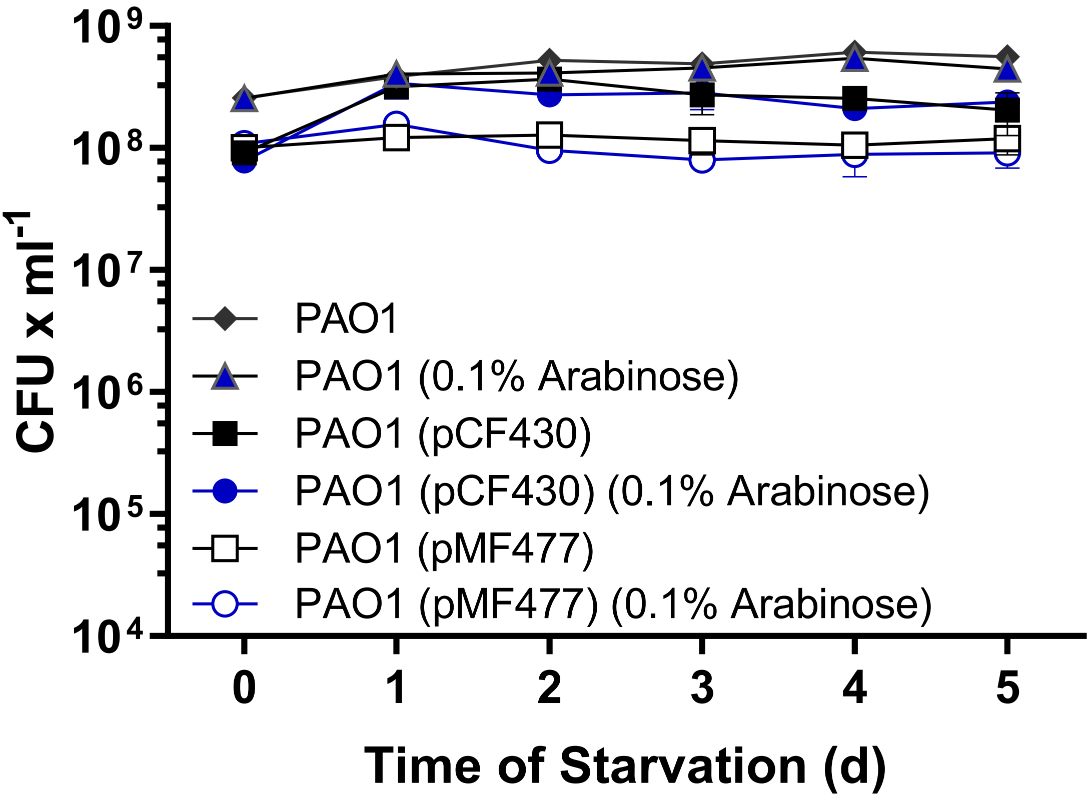

Figure S6. Effect of nutrient limitation on the viability of *P. aeruginosa* and *P. aeruginosa (*pCF430) and *P. aeruginosa* (pMF477), to determine the effect of the plasmids used in this study on survival of P. aeruginosa during starvation. Following growth to early stationary phase, cells were incubated in phosphate buffered saline with or without arabinose as indicated, for up to 5 days. The viability of the cells was determined as colony forming units. The results show no effect of the plasmids on the survival of *P. aeruginosa* during starvation, in the presence or absence of arabinose.

Table S1. Primers used in this study.

Gene amplification primers

RpoH Nhe 5’	CAGCTAGCGAGTTTCGTGAATCGGAGGATTCG
RpoH Hind 3’	GCAAGCTTGCGGGGTGCGGTTTTCGCG

Allelic exchange primers

RpoH HindIII 5’ Up	aaGCTTCGTCTTCGGCCTGACCATC
RpoH T7 Up R Acc	ctatagtgagtcgtattaggtaccGCGAATCCTCCGATTCACGAAAC.
RpoH HindIII 3’ Down	aagcTTGCGGATAGACCCAGGCCTCGC
RpoH T7 Down F Acc	ggtacctaatacgactcactatagTCGCCGCGCCTCGCGAAAACCGC.

RT-qPCR primers

acpLeftRT-PCR2	ACTCGGCGTGAAGGAAGAAG	Williamson, 2012
acpRightRT-PCR2	CGACGGTGTCAAGGGAGT	
PA4463-For (hpf)	GCCATTTCGACAAGATCACC	Williamson, 2012
PA4463-Rev (hpf)	GCTGCCGTTCGAGATACTTC	
PA3126-For (IbpA)	CTGTTCCGTCATTCCGTAGG	Williamson, 2012
PA3126-Rev (Ibpa)	GGCGATAACGATGCGATACT	
RpoH_F	CCGAACGCCTCTTCTATCAG	
RpoH_R	TTGGCGATATGAACAACGAA	
GroES_1F	GAGGAAGAGACCAAGACCGC	
GroES_1R	GTCCAGTACACGACCGGTAC	
PA1216_F	GACGACACCCTGGAGATCAT	
PA1216_R	GCTGGTGGAGAGGAAGAACA	
WapP_F	AGCCGCTGGTGTATTTCATC	
WapP_R	GTGGTACTTGGCCACCTTGT	

International Journal of
Molecular Sciences

MDPI

Table S2. Genes significantly downregulated in PAO1Δ*rpoH* (P_{BAD}-*rpoH*) by > 2-fold change and $p < 0.05$ following a 15-minute induction of *rpoH*.

**Gene**	**Function**	**Fold change**	***p*-value**
PA0035	Tryptophan synthase alpha chain	-2.2	1.3×10^{-2}
PA0179	Two-component response regulator	-2.0	2.6×10^{-2}
PA0423	Antibiotic sequester PasP	-2.4	2.0×10^{-3}
PA0460	hypothetical protein	-2.0	1.1×10^{-2}
PA0470	Ferrichrome receptor FiuA	-2.1	2.3×10^{-2}
PA0484	Similar to glycine cleavage system transcriptional repressor	-2.6	1.3×10^{-3}
PA0567	Conserved hypothetical protein	-2.4	3.6×10^{-3}
PA0586	Conserved hypothetical protein	-2.2	3.5×10^{-2}
PA0587	Contains a von Willebrand domain	-2.3	4.2×10^{-2}
PA0588	Possible response regulator	-2.1	1.1×10^{-3}
PA0672	Heme oxygenase	-2.7	1.7×10^{-2}
PA0707	Transcriptional regulator ToxR	-3.4	3.1×10^{-3}
PA0792	Propionate catabolic protein PrpD	-2.4	1.8×10^{-3}
PA0798	Phospholipid methyltransferase	-2.1	1.4×10^{-2}
PA0931	Ferric enterobactin receptor PirA	-2.2	2.1×10^{-2}
PA1041	Outer membrane protein precursor	-3.3	4.7×10^{-4}
PA1134	Hypothetical protein	-4.4	1.9×10^{-2}
PA1245	Alkaline protease secretion protein AprX	-2.6	7.4×10^{-4}
PA1246	Alkaline protease secretion protein AprD	-2.3	1.2×10^{-2}
PA1300	ECF sigma factor HxuI	-2.6	1.6×10^{-2}
PA1301	Anti-sigma factor HxuR	-2.1	1.2×10^{-2}
PA1650	Transporter	-2.3	1.4×10^{-3}
PA1713	Transcriptional regulator ExsA	-5.2	2.8×10^{-3}
PA1745	Hypothetical protein	-2.0	1.8×10^{-2}
PA1753	ATP binding/hydrolysis	-2.1	1.5×10^{-2}
PA1860	SAM-dependent methyltransferase	-2.8	1.7×10^{-3}
PA1871	LasA protease precursor	-2.0	1.4×10^{-2}
PA1888	Putative esterase/lipase	-3.1	4.0×10^{-2}
PA1911	Sigma factor regulator, FemR	-2.7	2.4×10^{-2}
PA2033	Siderophore-interacting FAD oxydoreductase	-3.1	5.8×10^{-3}
PA2034	SAM-dependent methyltransferase	-3.8	6.7×10^{-3}
PA2062	Pyridoxal-phosphate dependent enzyme	-2.5	2.2×10^{-2}
PA2174	Hypothetical protein	-2.6	3.4×10^{-2}
PA2204	Putative binding component of ABC transporter	-3.0	2.7×10^{-2}
PA2259	Transcriptional regulator PtxS	-2.0	3.5×10^{-4}
PA2364	Lip3, a membrane bound component of the type VI secretion system	-2.9	2.2×10^{-3}
PA2365	HsiB3, type VI secretion system	-4.2	8.9×10^{-3}

PA2366	HsiC3, type VI secretion system	-4.1	4.3×10^{-3}
PA2367	Hcp3, type VI secretion system	-4.3	3.1×10^{-2}
PA2368	HsiF3, type VI secretion system	-4.8	6.9×10^{-3}
PA2369	HsiG3, type VI secretion system	-2.1	2.2×10^{-2}
PA2370	HsiH3, type VI secretion system	-2.2	1.8×10^{-2}
PA2371	ClpA/B-type protease	-2.4	1.1×10^{-2}
PA2372	Hypothetical protein	-3.4	1.5×10^{-2}
PA2373	VgrG3, Phage base V domain-containing protein, type 6SS	-2.5	2.5×10^{-2}
PA2375	Hypothetical protein	-2.3	4.6×10^{-2}
PA2383	Transcriptional regulator	-2.8	4.8×10^{-3}
PA2384	Fur-like DNA binding domain	-3.8	8.0×10^{-4}
PA2385	3-oxo-C12-homoserine lactone acylase PvdQ	-5.1	1.4×10^{-3}
PA2386	L-ornithine N5-oxygenase	-3.7	1.6×10^{-3}
PA2389	PvdR	-3.9	7.0×10^{-3}
PA2390	PvdT	-5.5	3.1×10^{-4}
PA2391	Outer membrane protein precursor	-3.1	2.9×10^{-2}
PA2392	PvdP	-5.5	2.6×10^{-4}
PA2393	Putative dipeptidase	-5.8	1.2×10^{-2}
PA2394	PvdN	-5.8	2.3×10^{-3}
PA2395	PvdO	-5.5	1.3×10^{-3}
PA2396	Pyoverdine synthetase F	-3.8	2.5×10^{-4}
PA2397	Pyoverdine biosynthesis protein PvdE	-4.8	2.9×10^{-2}
PA2398	Ferripyoverdine receptor	-3.6	4.3×10^{-2}
PA2399	Pyoverdine synthetase D	-2.2	1.4×10^{-2}
PA2400	PvdJ, a nonribosomal peptide synthetase condensation module	-2.4	4.4×10^{-3}
PA2401	PvdJ, fused with previous	-2.9	8.0×10^{-3}
PA2402	Pyoverdine peptide synthetase	-4.4	1.6×10^{-3}
PA2404	FpvH	-2.4	2.6×10^{-3}
PA2405	FpvJ	-2.8	5.9×10^{-3}
PA2407	FpvC	-2.3	4.3×10^{-3}
PA2408	FpvD	-3.2	5.8×10^{-4}
PA2409	FpvE	-2.1	9.6×10^{-3}
PA2410	FpvF	-2.9	1.4×10^{-3}
PA2411	Thioesterase	-3.7	7.4×10^{-4}
PA2412	MbtH-like, a nonribosomal peptide synthetase partner domain	-3.3	2.8×10^{-3}
PA2413	L-2,4-diaminobutyrate:2-ketoglutarate 4-aminotransferase, PvdH	-6.3	8.0×10^{-4}
PA2415	Hypothetical protein	-2.6	9.1×10^{-3}
PA2424	PvdL	-7.1	7.5×10^{-4}
PA2425	PvdG	-7.5	4.7×10^{-4}
PA2427	hypothetical protein	-5.0	9.2×10^{-4}
PA2451	Putative esterase	-4.6	5.4×10^{-5}
PA2452	Putative esterase	-4.2	1.4×10^{-2}

PA2467	Anti-sigma factor FoxR	-2.6	1.2×10^{-3}
PA2468	ECF sigma factor FoxI	-2.1	4.1×10^{-2}
PA2572	Two-component response regulator	-2.1	2.9×10^{-3}
PA2573	Chemotaxis transducer	-2.7	8.6×10^{-6}
PA2746	Conserved hypothetical protein	-2.9	1.5×10^{-2}
PA2786	With GAF domain, putative photosensitive histidine kinase	-3.0	5.0×10^{-2}
PA2937	Hypothetical protein	-2.0	1.2×10^{-2}
PA3040	Hypothetical protein	-2.1	1.8×10^{-5}
PA3409	HasS, putative anti-sigma factor	-3.0	1.7×10^{-3}
PA3418	Leucine dehydrogenase	-2.1	2.5×10^{-2}
PA3451	Hypothetical protein	-2.1	3.3×10^{-2}
PA3688	Hypothetical protein	-2.1	2.5×10^{-2}
PA3691	DUF4398 domain-containing protein	-2.2	2.2×10^{-2}
PA3692	Lipotoxin F, LptF	-2.3	1.5×10^{-2}
PA3712	Hypothetical protein	-2.8	7.0×10^{-4}
PA3866	Pyocin S4	-2.6	3.2×10^{-2}
PA3900	FecR, anti-sigma regulator	-2.4	1.1×10^{-2}
PA4168	Second ferric pyoverdine receptor FpvB	-3.3	9.5×10^{-4}
PA4227	Transcriptional regulator PchR	-2.4	2.6×10^{-2}
PA4296	Two-component response regulator, PprB	-2.0	2.0×10^{-3}
PA4390	Putative outer membrane porin	-2.8	8.7×10^{-3}
PA4467	Putative metal transporter	-5.8	1.2×10^{-3}
PA4468	Superoxide dismutase	-3.7	4.4×10^{-3}
PA4469	A TPR-repeat protein	-3.2	2.4×10^{-2}
PA4471	Hypothetical protein	-4.2	4.5×10^{-2}
PA4570	Hypothetical protein	-3.7	3.7×10^{-3}
PA4607	Hypothetical protein	-2.5	1.2×10^{-2}
PA4614	Conductance mechanosensitive channel	-2.1	3.2×10^{-2}
PA4710	Heme/Hemoglobin uptake outer membrane receptor PhuR precursor	-5.3	1.5×10^{-3}
PA4895	Transmembrane sensor	-2.2	3.0×10^{-2}
PA4896	Sigma-70 factor, ECF subfamily	-2.8	2.1×10^{-2}
PA5150	Short-chain dehydrogenase	-2.3	8.1×10^{-3}
PA5173	Carbamate kinase	-2.3	9.5×10^{-4}
PA5178	Putative potassium binding protein	-2.2	9.3×10^{-3}
PA5424	Hypothetical protein	-2.4	2.4×10^{-3}
PA5528	Hypothetical protein	-2.5	7.6×10^{-3}
